# Spiked proteomic standard dataset for testing label-free quantitative software and statistical methods

**DOI:** 10.1016/j.dib.2015.11.063

**Published:** 2015-12-17

**Authors:** Claire Ramus, Agnès Hovasse, Marlène Marcellin, Anne-Marie Hesse, Emmanuelle Mouton-Barbosa, David Bouyssié, Sebastian Vaca, Christine Carapito, Karima Chaoui, Christophe Bruley, Jérôme Garin, Sarah Cianférani, Myriam Ferro, Alain Van Dorssaeler, Odile Burlet-Schiltz, Christine Schaeffer, Yohann Couté, Anne Gonzalez de Peredo

**Affiliations:** a ProFi, Proteomic French Infrastructure, France; b CNRS UMR5089 Institut de Pharmacologie et de Biologie Structurale, 205 Route de Narbonne, 31077 Toulouse, France; c Université de Toulouse, 118 Route de Narbonne, 31077 Toulouse, France; d CEA, DSV, iRTSV, Laboratoire de Biologie à Grande Echelle, Grenoble F-38054, France; e INSERM U1038, Grenoble F-38054, France; f Université Grenoble, F-38054, France; g Laboratoire de Spectrométrie de Masse BioOrganique (LSMBO), IPHC, Université de Strasbourg, CNRS, UMR7178, 25 Rue Becquerel, 67087 Strasbourg, France

## Abstract

This data article describes a controlled, spiked proteomic dataset for which the “ground truth” of variant proteins is known. It is based on the LC-MS analysis of samples composed of a fixed background of yeast lysate and different spiked amounts of the UPS1 mixture of 48 recombinant proteins. It can be used to objectively evaluate bioinformatic pipelines for label-free quantitative analysis, and their ability to detect variant proteins with good sensitivity and low false discovery rate in large-scale proteomic studies. More specifically, it can be useful for tuning software tools parameters, but also testing new algorithms for label-free quantitative analysis, or for evaluation of downstream statistical methods. The raw MS files can be downloaded from ProteomeXchange with identifier PXD001819. Starting from some raw files of this dataset, we also provide here some processed data obtained through various bioinformatics tools (including MaxQuant, Skyline, MFPaQ, IRMa-hEIDI and Scaffold) in different workflows, to exemplify the use of such data in the context of software benchmarking, as discussed in details in the accompanying manuscript [1]. The experimental design used here for data processing takes advantage of the different spike levels introduced in the samples composing the dataset, and processed data are merged in a single file to facilitate the evaluation and illustration of software tools results for the detection of variant proteins with different absolute expression levels and fold change values.

**Specifications Table**

**Table:** 

Subject area	*Bioinformatics for proteomics*
More specific subject area	*Label-free quantification of proteomic data*
Type of data	*MS data, table and figures*
How data was acquired	*Mass spectrometry (LTQ-Velos data)*
Data format	*Raw+Excel file (data processed with different bioinformatics workflows)*
Experimental factors	*Data was obtained from the MS analysis of a proteomic standard formed of a fixed background (yeast cell lysate) spiked with 9 different amounts of the UPS1 standard mixture (Sigma).*
Experimental features	*Samples corresponding to the 9 different concentrations of UPS1 spiked in yeast lysate were reduced and alkylated, digested with trypsin, and analyzed in triplicate by nanoLC-MS/MS on a LTQ-Velos Orbitrap. Different workflows were applied for data processing and label-free quantification of proteins in pairwise comparison of different UPS1 concentrations.*
Data source location	*Toulouse, France*
*Grenoble, France*
*Strasbourg, France*
Data accessibility	*All raw MS data is deposited in the ProteomeXchange repository with the identifier PXD001819. Protein quantitative metrics obtained from different bioinformatics workflow are provided in Supplementary Table*^1^.

## 1 Value of the data

We provide a standard proteomic dataset based on a highly complex sample (yeast lysate) spiked with different levels of a second calibrated protein mixture of medium complexity (UPS1 standard, 48 proteins), that can be used to statistically evaluate label-free approaches for detection of differentially abundant proteins.

• This spiked dataset can be used for benchmarking and comparing softwares for label-free quantification.
• It may represent a convenient tool for users who want to optimize the tuning and find the best parameters for a particular software.
• It can be useful for developers in order to test algorithms and improve the extraction of intensity metrics for protein quantitation.
• It could also be applied to the evaluation of post-processing steps (normalization, imputation of missing values) and statistical methods.

## 2 Data and experimental design

**Fig. 1:**
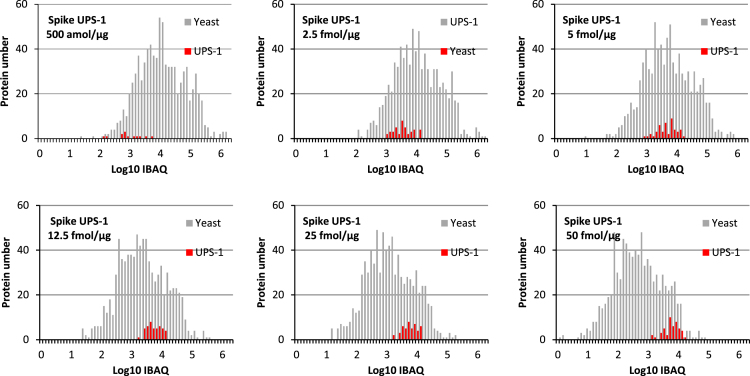
illustration of the absolute abundance of spiked proteins compared to the yeast background in the 6 last samples of the dataset. Absolute abundances were estimated using the iBAQ metric calculated by MaxQuant in workflows 6 and 7 (see below for the details of the workflows).

We provide a dataset composed of raw MS files corresponding to the analysis of a series of yeast cell lysate samples spiked with different amounts of an equimolar mixture of 48 recombinant proteins (Sigma UPS1). The 9 different samples were analyzed in triplicate on a LTQ-Velos Orbitrap and the resulting 27 raw files can be downloaded from ProteomeXchange using the identifier PXD001819. The spiked UPS1 proteins can be easily identified after database search and constitute the “ground truth” panel of differentially abundant proteins in quantitative pairwise comparison of samples from the dataset. Conversely, the background of yeast proteins should remain invariant after quantitative comparison of these samples. As UPS1 proteins constitute a very minor proportion of the global proteome for each sample (see Fig. 1 showing the histogram of iBAQ values for yeast background an UPS1 proteins respectively in the different samples), the samples can in principle be used to simulate a biological situation where only a minor part of the protein population undergoes expression changes, and data can be normalized based on the median of intensity values for the global protein population. Nevertheless, the spiked mixture is relatively complex (48 proteins) and can be used to statistically approximate the sensitivity of the analytical and bioinformatics workflows, by calculating the proportion of UPS1 proteins truly found as showing differential signals.

As a proof of principle of the potential utility of this dataset, we also provide some processed data, in Excel format (Supplementary Table 1), obtained from different bioinformatics workflows combining tools for database search, protein validation, and label-free quantification. These workflows are described in details in [1] and in the Materials and Methods section below, and include MFPaQ [2], [3], Irma/Heidi, Scaffold, MaxQuant [4], [5], [6] and Skyline [7], [8] as tools to extract quantitative metrics. The quantitative value extracted for each protein is either a total spectral count obtained after the validation step, or a protein intensity value obtained from the MS signal intensity of associated peptides. The Excel file provided here (Supplementary Table 1) is composed of 8 different sheets containing the quantitative data from the 8 different workflows tested (4 spectral count and 4 MS intensity based workflows).

**Fig. 2:**
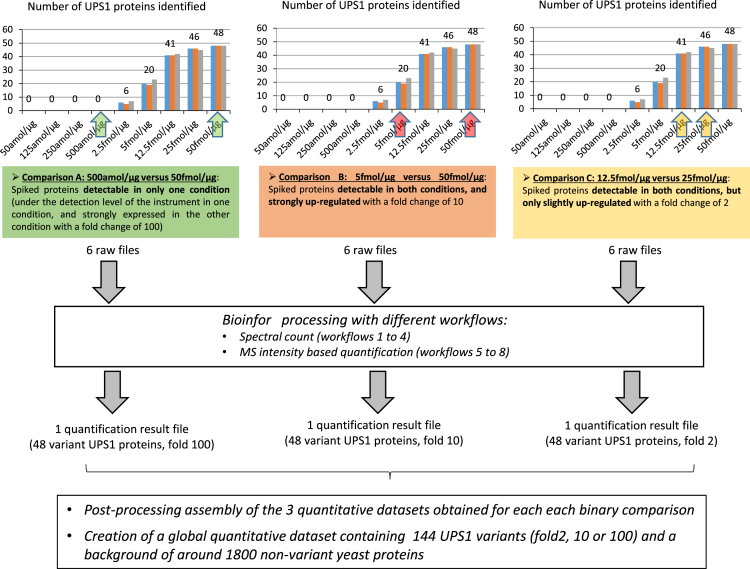
Experimental design of the data processing workflow.

The experimental design of the data processing is illustrated in Fig. 2. Among the 9 spiked samples analyzed, we selected 5 of them in order to perform different pairwise quantitative comparison of samples, trying to mimic distinct biochemical situations (comparisons A, B and C: detection in only one condition; high fold change; moderate fold change). The quantitative outputs obtained from these pairwise comparisons were then combined, in order to reconstruct a simulated dataset containing true-positive hits with different intensities and fold change values, and to illustrate the performances of the bioinformatic and statistical methods in a more comprehensive way. Each Excel sheet in Supplementary Table 1 corresponds to this mixed dataset, for a particular bioinformatic workflow.

## 3 Materials and methods

*Sample preparation*. A yeast cell lysate was prepared in 8 M urea/0.1 M ammonium bicarbonate buffer, protein concentration was adjusted at 8 µg/µL after Bradford assay, and this lysate was used to resuspend and perform a serial dilution of the UPS1 standard mixture (Sigma). Twenty µL of each of the resulting samples, corresponding to 9 different spiked levels of UPS1 (respectively 0.05–0.125–0.250–0.5–2.5–5–12.5–25–50 fmol of UPS1/µg of yeast lysate), were reduced with DTT and alkylated with iodoacetamide. The urea concentration was lowered to 1 M by dilution, and proteins were digested in solution by addition of 2% of trypsin overnight. Enzymatic digestion was stopped by addition of TFA (0.5% final concentration).

*NanoLC-MS/MS analysis*. Samples (2 µg of yeast cell lysate+different spiked level of UPS1) were analyzed in triplicate by nanoLC-MS/MS using a nanoRS UHPLC system (Dionex, Amsterdam, The Netherlands) coupled to an LTQ-Orbitrap Velos mass spectrometer (Thermo Fisher Scientific, Bremen, Germany). 2 µL of each sample were loaded on a C-18 precolumn (300 µm IDx5 mm, Dionex) at 20 µL/min in 5% acetonitrile, 0.05% TFA. After 5 min desalting, the precolumn was switched online with the analytical C-18 column (75 µm IDx15 cm, in-house packed with C-18 Reprosil) equilibrated in 95% solvent A (5% acetonitrile, 0.2% formic acid) and 5% solvent B (80% acetonitrile, 0.2% formic acid). Peptides were eluted using the following gradient of solvent B at 300 nL/min flow rate: 5–25% gradient during 75 min; 25–50% during 30 min; 50–100% during 10 min. The LTQ-Orbitrap Velos was operated in data-dependent acquisition mode with the XCalibur software. Survey scan MS were acquired in the Orbitrap on the 300–2000 m/z range with the resolution set to a value of 60,000. The 20 most intense ions per survey scan were selected for CID fragmentation and the resulting fragments were analyzed in the linear trap (LTQ). Dynamic exclusion was employed within 60 s to prevent repetitive selection of the same peptide.

*MS data processing*. The dataset was processed according to different workflows listed in Table 1 from Ref. [1], consisting in the following steps: peaklist generation, database search, validation of the identified proteins and extraction of quantitative metric (spectral count or MS signal). According to the different tools used for each step, 8 distinct workflows were evaluated. The same databases were used for peptide identifications: yeast database from UniprotKB (S_cerevisiae_ 20121108.fasta, 7798 sequences) and a compiled database containing the UPS1 human sequences (48 sequences).

*Bioinformatic workflow* 1*: ExtractMSn/Mascot/MFPaQ/Spectral Counting*. The Mascot Daemon software (version 2.4; Matrix Science, London, UK) was used to perform database searches, using the Extract_msn.exe macro provided with Xcalibur (version 2.0 SR2; Thermo Fisher Scientific) to generate peaklists. Parameters used for creation of the peaklists were: parent ions in the mass range 400–4500, no grouping of MS/MS scans, and threshold at 1000. Peaklists were submitted to Mascot database searches (version 2.4.2). ESI-TRAP was chosen as the instrument, trypsin/P as the enzyme and 2 missed cleavages were allowed. Precursor and fragment mass error tolerances were set at 5 ppm and 0.8 Da, respectively. Peptide variable modifications allowed during the search were: acetyl (Protein N-ter), oxidation (M), whereas carbamidomethyl (C) was set as fixed modification. To calculate the false discovery rate (FDR), the search was performed using the “decoy” option in Mascot. Validation was performed with an in-house developed module associated to MFPaQ [2] (http://mfpaq.sourceforge.net/), based on the target-decoy strategy, as described before [3]. Briefly, FDR at peptide level was calculated as described in [9] and set at 5% by adjusting peptide p-value threshold. Validated peptides were assembled into protein groups following the principle of parsimony (Occam׳s razor) [10]. Protein groups were then validated to obtain a FDR of 1% at the protein level, by adjusting the threshold on a protein group score defined as the sum of peptide score offsets (difference between each peptide Mascot score and its homology or identity threshold). The total spectral count metric was extracted for each protein group by MFPaQ in each analytical run.

*Workflow* 2*: Andromeda/MaxQuant/Spectral Counting*. Acquired MS data were processed using MaxQuant version 1.3.0.5 [4]. Derived peak lists were submitted to the Andromeda search engine [6]) (www.maxquant.org). For database searches, the precursor mass tolerance was set to 20 ppm for first searches and 6 ppm for main Andromeda database searches. The fragment ion mass tolerance was set to 0.5 Da. Trypsin/P was chosen as the enzyme and 2 missed cleavages were allowed. Oxidation of methionine and protein N-terminal acetylation were defined as variable modifications, and carbamidomethylation of cysteine was defined as a fixed modification. Minimum peptide length was set to six amino acids. Minimum number of unique peptides was set to one. Maximum FDR – calculated by employing a reverse database strategy – were set to 1% for peptides and proteins. Proteins identified as “reverse” and “only identified by site” were discarded from the list of identified proteins. In this particular workflow, total spectral count for each validated protein group was computed from msms.txt table.

*Workflow* 3*: Mascot Distiller/Mascot/IRMa-hEIDI/Spectral Counting*. Data were processed automatically using Mascot Distiller software (version 2.4.3.0, Matrix Science). ESI-TRAP was chosen as the instrument, trypsin/P as the enzyme and 2 missed cleavages were allowed. Precursor and fragment mass error tolerances were set at 5 ppm and 0.8 Da, respectively. Peptide variable modifications allowed during the search were: acetylation (Protein N-ter), oxidation (M), whereas carbamidomethyl (C) was set as fixed modification. The IRMa software v1.31 [11] was used to filter the results. Filters used were: (1) peptides whose score≥query homology threshold (*p*<0.5) and rank≤1 are marked as significant; (2) Single match per query filter was: Move to ambiguous all peptides which are not assigned to best protein for this query (best is higher protein score); (3) FDR seeker filter: seek a 1% FDR based on score filtering; (4) Accession filter: Delete proteins coming from reverse database; (5) Specific peptide filter: accept only protein hits whose specific peptides count >=1. The filtered results were then compiled and structured within dedicated relational Databases and a homemade tool (hEIDI) was used for the compilation, grouping and comparison of the proteins from the different samples, analytical replicates and conditions to compare (Hesse et al., in preparation). In such workflow, total spectral count values calculated for each protein groups are used for quantification.

*Workflow* 4*: ExtractMSn/Mascot/Scaffold/Spectral Counting*. Peaklists generation and protein identifications were made as detailed in workflow 1. Mascot results were loaded into the Scaffold software (Version 3.6.5, Proteome Software, Portland, USA). To minimize false positive identifications, results were subjected to very stringent filtering criteria as follows. For the identification of proteins, a Mascot ion score had to be minimum 30 and above the 95% Mascot significance threshold ("Identity score"). The target-decoy database search allowed us to control and estimate the false positive identification rate of our study, and the final catalog of proteins presented an estimated false discovery rate (FDR) below 5%. The spectral count metric used for quantitation corresponds to the Unweighted Spectrum Count values in Scaffold.

*Workflow* 5*: ExtractMSn/Mascot/MFPaQ/MS Signal analysis*. The first steps (peaklist creation, database search, validation) were the same than in workflow 1. Quantification of proteins was then performed using the label-free module implemented in the MFPaQ v4.0.0 software, as previously described [3], [12]. Briefly, the software uses the validated identification results and retrieves the XIC of the identified peptide ions in the corresponding raw nanoLC-MS files, based on their experimentally measured RT and monoisotopic *m*/*z* values. Peptide ions identified in all the samples to be compared are used to build a retention time matrix and re-align in time LC-MS runs. For peptides not identified by MS/MS in a particular run, this re-alignment matrix is used to perform cross-assignment and extract their XIC signal starting from a predicted RT. Normalization across conditions is performed based on the median of XIC area ratios for all the extracted peptide ions. Protein quantification is based on a protein abundance index calculated as the average of XIC area values for at most three intense reference tryptic peptides per protein.

*Workflow* 6 *and* 7*: Andromeda/MaxQuant/MS Signal analysis*. The first steps (database search with Andromeda and validation) were the same as in workflow 2. For quantification purposes, either Intensities (workflow 6) or LFQ [13] (workflow 7) calculated by MaxQuant were used. The LFQ metric, as described in [13], is derived from the raw intensities by the MaxLFQ algorithm, which uses a specific normalization procedure, as well as a particular aggregation method to calculate protein intensities, by taking into account, for each protein, all the peptide ratios measured in all pairwise comparisons of the different quantified samples. “Match between run” time window was set to 2 min. For LFQ quantification, only protein ratios calculated from at least two unique peptides ratios (min LFQ ratio count=2) were considered for calculation of the LFQ protein intensity.

*Workflow* 8*: Mascot Distiller/Mascot/Skyline/MS Signal analysis*. Peaklist creation was performed with Mascot Distiller as described in workflow 3, then database searches were performed with Mascot and validated with Scaffold as described for workflow 4. XIC signal corresponding to all validated peptides were extracted using the Skyline software (Skyline version v2.5, daily updates of April 2014, https://skyline.gs.washington.edu). This method was well described by Schilling et al (Schilling et al. MCP, 2012). Total areas, corresponding to the sum of the 3 extracted isotopes areas, were used for statistical analysis.

*Statistical analysis*. For pairwise comparisons of samples spiked at different concentrations of UPS1, same statistical tests and fold change criteria were applied to the quantitative data obtained from each workflow, as follows:

When working on spectral count metrics (workflows 1-2-3-4), a beta-binomial test was performed based on triplicate MS/MS analyzes. p-values were calculated with the software package BetaBinomial_1.2 [14] implemented in R. Fold change was calculated as ratio of average spectral counts from both conditions. For proteins absent in all replicates of one specific condition, their spectral count values were modified by adding 1 spectrum to all 6 samples in order to be able to calculate a fold change for these particular proteins.

When working on MS signal intensity-based metrics (workflows 5–6–7–8), proteins were filtered out if they were not quantified in at least all replicates from one condition. Missing protein intensity values were replaced by a constant value calculated independently for each sample as the 5-percentile value of the total population. A welch *t*-test (two-tailed *t*-test, unequal variances) based on triplicate MS analyzes was then performed on log2 transformed values using the Perseus toolbox (version 1.4.0.11; http://141.61.102.17/perseus_doku). Criteria used to classify the proteins were the Welch *t*-test difference calculated by Perseus (difference between the two compared conditions of the mean log2 transformed value for triplicate MS/MS analyzes), and the Welch *t*-test *p*-value.

*Construction of the mixed dataset and plot of the ROC curves for each workflow*

For each workflow, quantitative outputs from the 3 pairwise comparisons described in Fig. 2 were merged in a single Excel table. This composite table is shown for each of the 8 tested workflows in a separate sheet in Supplementary Table 1. The first column indicates the origin of the quantitative values: comparison A (50Vs0.5), comparison B (50Vs5) or comparison C (25Vs12.5). The second column indicates whether the protein originate from the background (yeast) or from the spiked mixture (UPS). These UPS1 proteins are highlighted in green, red, or yellow according to the comparison in which they were quantified (A, B, and C respectively). Following columns indicate:

1. Identification data reported by the different tools after database search and validation (fasta header, protein ID, number of peptides, score, etc…)
2. Raw quantitative data (6 columns: 2 conditions, 3 technical replicates) containing missing values
3. Transformed data (for MS intensity based workflows 5–8) after imputation of missing values as described above, and logarithmic transformation.
4. Fold change values and statistical test values as calculated by the BetaBinomial package (spectral count) or by Perseus (MS intensity values). In the latter case, a *z*-score was also calculated for each protein as follows:

*z*-score={(Welch t-test difference)- Median [(Welch *t*-test difference) for all quantified proteins] }/Standard deviation [(Welch *t*-test difference) for all quantified proteins]

To classify proteins as variant and non-variant and plot ROC curves, different combinations of criteria were tested:

1. for spectral count workflows: |log2 fold change|>x (from 0.8–3) and *p*-value<*y* (from 0.05 to 0.0001)
2. for MS intensity based workflows: |welch *t*-test difference|>x (from 0 to 7) and *p*-value<*y* (from 0.3 to 0.0001)

Proteins classified as variant according to these criteria were counted as true-positive (TP) if they were UPS1, and false positive (FP) if they were from yeast. Proteins classified as non-variant according to these criteria were counted as true-negative (TN) if they were from yeast, and false-negative (FN) if they were UPS1. Sensitivity of the workflow was calculated as TP/144 (taking into account 3×48=144 real differentially abundant UPS1 proteins after mixing of the 3 quantitative outputs) and False Discovery Proportion (FDP) was calculated as FP/(TP+FP).

**Fig. 3:**
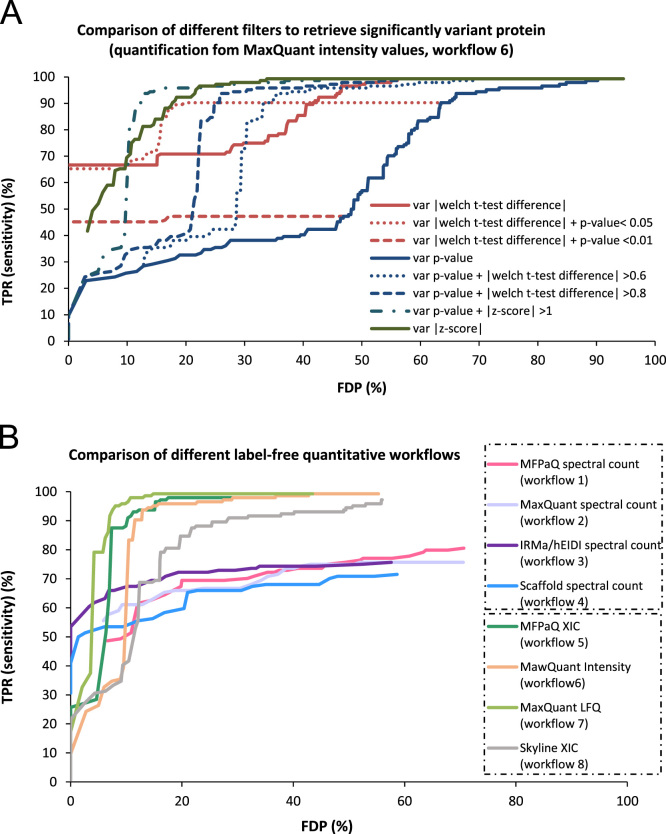
ROC curves plotted from the dataset to compare filtering criteria (A) or bioinformatics workflows (B). A/sensitivity-FDP curves were plotted for the data obtained from workflows 6 (quantification based on MaxQuant intensity values) by varying either the |Welch *t*-test difference| threshold (red), the |*z*-score| threshold (green) or the Welch *t*-test *p*-value threshold (blue). The Welch *t*-test difference, *z*-score or *p*-value were used respectively as a unique criterion to classify the proteins (full line curves), or a combinations of these filters were applied to improve the classification (dotted line curves). B/Overlaid ROC curves for the different bioinformatics workflows: proteins were classified as variant by filtering on the *p*-value thresholds, combined to a fixed |log2(fold change)| threshold of 1 for spectral count workflows (1–4) and to a fixed |*z*-score| threshold of 1 for MS intensity based workflows (5–8).

Fig. 3 shows ROC curves (sensitivity versus FDP) which illustrate how the dataset can be useful for selecting the most efficient classification filters (Fig. 3A) or for the comparison of software tools (Fig. 3B).

## Footnotes

Appendix A Supplementary data associated with this article can be found in the online version at 10.1016/j.dib.2015.11.063.
